# Innovations in Cutaneous Oncology and Dermatologic Surgery: From Margin Control to Integrated Precision Oncology

**DOI:** 10.3390/cancers18040670

**Published:** 2026-02-18

**Authors:** Maria Goreti Baião Catorze, Paulo Filipe

**Affiliations:** 1 Department of Dermatology, Hospital Egas Moniz—ULS Lisboa Ocidental, 1349-019 Lisbon, Portugal; 2 Department of Dermatology, Centro Hospitalar Universitário de Lisboa Norte, Faculty of Medicine, University of Lisbon, 1649-019 Lisbon, Portugal; paulolealfilipe@gmail.com

**Keywords:** cutaneous oncology, dermatologic surgery, Mohs micrographic surgery, precision oncology, immunotherapy, targeted therapy, multidisciplinary care

## Abstract

Cutaneous oncology is undergoing a major transformation driven by advances in tumour biology, immunotherapy, targeted agents, and locoregional technologies. Although surgery remains the cornerstone of curative treatment for most skin cancers, an increasing proportion of patients present with high-risk, locally advanced, recurrent, or biologically aggressive disease that challenges a surgery-only paradigm. In this evolving landscape, dermatologic surgery has transitioned from a standalone intervention to a central component of integrated precision oncology. This narrative review provides a clinically oriented synthesis of recent innovations in cutaneous oncology and dermatologic surgery, with a focus on risk-adapted surgical decision-making and multidisciplinary treatment sequencing. We examine the evolving role of margin-controlled and function-preserving techniques, particularly Mohs micrographic surgery, and define clinical scenarios in which standard excision, Mohs surgery, radiotherapy, systemic therapy, or combined approaches are preferred. Quantitative outcome data from pivotal trials are incorporated where available, including local control, recurrence risk, response rates, and survival outcomes. Across major cutaneous malignancies—basal cell carcinoma, cutaneous squamous cell carcinoma, melanoma, and selected rare tumours—we discuss how targeted therapies, immune checkpoint inhibitors, radiotherapy, and locoregional treatments are increasingly integrated with surgery in neoadjuvant, adjuvant, consolidative, and salvage settings. Particular attention is given to treatment-related toxicities, patient selection, and the implications of systemic therapy on surgical timing, reconstruction, and morbidity. High-risk populations, including immunosuppressed patients, are specifically addressed. By outlining adaptive therapeutic algorithms and emphasizing multidisciplinary collaboration, this review highlights the expanding role of the dermatologic surgeon in modern oncology. Surgery remains indispensable for local control and cure; however, its greatest impact now lies in strategic integration with systemic and locoregional therapies to optimise oncologic outcomes, preserve function, and deliver personalised, patient-centred care.

## Simple Summary

Skin cancer treatment has evolved beyond surgery alone. While surgical excision remains the cornerstone of cure for most tumours, advances in targeted therapy, immunotherapy, and locoregional treatments have transformed care for complex and advanced cases. Dermatologic surgery is now integrated within multidisciplinary precision oncology, where treatment sequencing combines surgery with systemic and radiotherapeutic approaches to optimize outcomes and preserve function. This review summarizes recent innovations in cutaneous oncology and explains how modern dermatologic surgeons play a central role in coordinating personalized, multimodal cancer care.

## 1. Introduction

Cutaneous oncology has entered a phase of accelerated transformation driven by demographic changes, advances in tumour biology, and the rapid expansion of systemic and locoregional therapies. While surgical excision remains the definitive curative modality for the majority of skin cancers, an increasing proportion of patients now present with tumours that challenge the traditional paradigm of surgery alone. These include locally advanced disease, aggressive histologic subtypes, recurrent tumours after multiple interventions, and malignancies arising in immunosuppressed or frail populations.

Historically, dermatologic surgery developed in parallel with cutaneous oncology, achieving high cure rates through progressively refined excisional and reconstructive techniques. In the modern era, however, the advent of therapies capable of modifying tumour biology, immune surveillance, and disease trajectory has fundamentally reshaped treatment pathways. Rather than displacing surgery, these innovations have repositioned it within a multimodal and adaptive framework. In this setting, excision is strategically combined with systemic therapy, radiotherapy, and locoregional approaches.

Despite the growing number of reviews addressing individual aspects of cutaneous oncology—such as surgical techniques, immunotherapy, or targeted systemic treatments—there remains a lack of integrative, clinically focused analyses that explicitly address how these modalities should be combined in everyday practice. Existing reviews often describe therapeutic advances in isolation, with limited guidance on risk-adapted surgical decision-making, treatment sequencing, or the practical implications of systemic and locoregional therapies for dermatologic surgeons.

The objective of this review is therefore threefold:

(i) To define contemporary, risk-adapted criteria guiding surgical decision-making in cutaneous oncology, including when margin-controlled surgery is preferred over conventional excision;
(ii) To clarify when and how systemic therapies, radiotherapy, and locoregional approaches should be integrated with surgery across major cutaneous malignancies; and
(iii) To propose adaptive, clinically applicable treatment algorithms that reflect real-world multidisciplinary care [1,2,3,4].

## 2. Methods

This review was conducted as a narrative, clinically oriented synthesis of current evidence on basal cell carcinoma (BCC). Relevant publications were identified through searches in PubMed/MEDLINE, Scopus, and Web of Science. The search focused primarily on articles published in English within the last 10–15 years, while seminal earlier studies were included when historically or scientifically relevant.

Study selection was guided by clinical relevance, methodological robustness, and contribution to understanding BCC pathogenesis, risk stratification, surgical management, and systemic therapies. Priority was given to randomized clinical trials, meta-analyses, prospective cohort studies, and international consensus guidelines.

As this manuscript was designed as a narrative review, no formal predefined inclusion or exclusion criteria or systematic screening protocol were applied. The objective was not to achieve exhaustive quantitative synthesis, but rather to provide a structured, integrative, and clinically meaningful overview of current advances in BCC management.

Given the broad and multidisciplinary scope of this topic—encompassing epidemiology, molecular biology, surgical innovation, systemic therapies, and translational advances—a narrative approach was considered more appropriate than a systematic review in order to deliver an integrated and practice-oriented perspective.

## 3. Dermatologic Surgery as the Backbone of Cutaneous Oncology

### 3.1. From Conventional Excision to Precision Surgery

Surgical excision remains the first-line treatment for most basal cell carcinomas (BCCs), cutaneous squamous cell carcinomas (cSCCs), and primary melanomas. However, the concept of “adequate margins” has evolved from fixed numerical recommendations toward risk-adapted, biologically informed decision-making. Low-risk tumours are generally managed with standard excision, whereas high-risk lesions—defined by size, anatomic location, histologic subtype, recurrence, or perineural invasion—require enhanced margin assessment strategies. This evolution reflects a broader shift from purely technical surgery toward precision oncologic intervention, in which surgical planning is increasingly tailored to tumour biology and risk profile [1,2,3,4].

### 3.2. Mohs Micrographic Surgery as a Paradigm of Innovation

Mohs micrographic surgery (MMS) represents one of the most impactful innovations in dermatologic surgery. By enabling complete circumferential peripheral and deep margin assessment, MMS achieves superior local control while preserving maximal amounts of healthy tissue. Over time, its indications have expanded to include aggressive BCC subtypes (such as morpheaform, infiltrative, and basosquamous variants), high-risk cSCC, recurrent tumours, lesions in anatomically critical regions (including periorbital, nasal, and auricular areas), and selected rare adnexal malignancies [1,2,3,4].

Importantly, MMS is increasingly integrated within multimodal treatment strategies, including systemic and adjuvant therapies, particularly in advanced disease. This integration reinforces its role as part of a therapeutic sequence rather than an isolated surgical intervention [1,2,3,4].

### 3.3. Reconstruction as an Oncologic Strategy

Precision surgery in BCC aims not only to achieve complete tumour clearance but also to optimize functional and aesthetic outcomes, particularly in anatomically critical areas. Mohs micrographic surgery (MMS) has consistently demonstrated superior margin control compared to conventional excision in high-risk BCC, with reported 5-year recurrence rates ranging from approximately 1–3% for primary tumours and 5–7% for recurrent tumours, generally lower than those observed with standard excision in comparable high-risk settings.

Beyond margin assessment, precision surgery integrates preoperative risk stratification, intraoperative histological mapping, and reconstructive planning tailored to potential adjuvant treatments. In high-risk tumours with features such as perineural invasion or close margins, reconstructive strategies may be deliberately selected to remain compatible with postoperative radiotherapy, avoiding excessive tissue bulk or flap configurations that could compromise radiation delivery.

**Figure 1:**
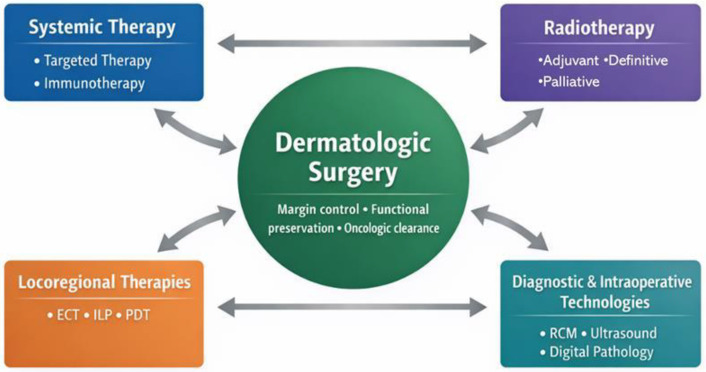
Dermatologic surgery as the central pillar of integrated precision oncology in cutaneous malignancies.

Reconstruction following oncologic surgery is no longer regarded as a purely aesthetic concern. Contemporary reconstructive planning must anticipate the potential need for adjuvant radiotherapy, the likelihood of recurrence, requirements for long-term surveillance, and preservation of critical functions such as vision, speech, and mastication. Integration of reconstruction into oncologic planning represents a key component of precision surgery, aligning surgical technique with durable disease control, functional preservation, and patient-reported quality-of-life outcomes [1,2,3,4] (Figure 1).

A risk-adapted framework integrating tumour characteristics and therapeutic strategy is summarized in Table 1.

This outcome-oriented, individualized approach reflects the ongoing evolution of BCC management toward biologically informed and function-preserving strategies.

## 4. Basal Cell Carcinoma: From Surgical Excellence to Integrated Targeted and Immunomodulatory Therapies

Basal cell carcinoma (BCC) is the most common cutaneous malignancy and, in the vast majority of cases, remains a surgically curable disease. Standard surgical excision and Mohs micrographic surgery provide excellent local control and remain the treatments of choice for most primary and recurrent tumours. Nevertheless, a subset of patients develops locally advanced or metastatic disease in which surgery and radiotherapy are no longer feasible or would result in unacceptable functional or aesthetic morbidity. In these cases, systemic medical therapies have fundamentally expanded the therapeutic landscape of advanced BCC [5,6,7,8].

### 4.1. Surgical Excellence and Limitations of Surgery Alone in Advanced BCC

While most BCCs are cured surgically, locally advanced disease may be characterised by deep infiltration, extensive tumour size, critical anatomic involvement, or repeated recurrence. In such scenarios, surgery alone may be technically unfeasible or associated with unacceptable morbidity, highlighting the need for alternative or adjunctive treatment strategies within a multimodal framework [5,6,7,8].

### 4.2. Targeted Therapy: Hedgehog Pathway Inhibition and Surgical Strategy

The identification of aberrant Hedgehog signalling as a central molecular driver of BCC pathogenesis has fundamentally altered disease management. Smoothened (SMO) inhibitors, including vismodegib and sonidegib, are approved for the treatment of locally advanced and metastatic BCC in patients who are not candidates for surgery or radiotherapy. Hedgehog pathway inhibitors (HHIs) have demonstrated significant tumour regression, disease control, and, in selected cases, prolonged survival, with particular clinical impact in patients with extensive facial tumours, multiple recurrences, or genetic predisposition syndromes such as basal cell nevus syndrome.

From a surgical perspective, HHIs have enabled neoadjuvant or downstaging strategies, including tumour volume reduction, conversion from unresectable to resectable disease, facilitation of margin-controlled surgery, and reduction in defect size and reconstructive complexity. However, resistance, adverse effects, incomplete responses, and tumour persistence after treatment discontinuation underscore the continued necessity of surgical expertise and definitive clearance whenever feasible. Increasingly, optimal care involves response-adapted surgery, timed to maximise therapeutic benefit.

Long-term treatment with HHIs is frequently limited by adverse effects such as muscle cramps, dysgeusia, alopecia, fatigue, and weight loss, leading to treatment interruption or discontinuation in a substantial proportion of patients [5,6,7,8].

### 4.3. Immunotherapy in Advanced Basal Cell Carcinoma

Immune checkpoint inhibition has emerged as an additional therapeutic option for patients with advanced BCC who progress on or are intolerant to Hedgehog pathway inhibitors. The anti–PD-1 antibody cemiplimab has demonstrated clinically meaningful activity in this setting, offering durable responses in a subset of heavily pretreated patients. Although immunotherapy does not replace surgery in resectable disease, its availability further reinforces the transition toward an integrated oncologic model in which systemic therapies complement surgical intervention in selected advanced cases [5,6,7,8].

### 4.4. Radiotherapy and Multimodal BCC Management

Radiotherapy remains an important modality for selected BCCs, particularly when surgery is contraindicated. Recent innovations include refined patient selection, improved fractionation schedules, integration with systemic therapy, and coordinated reconstructive planning to mitigate late tissue effects. Within a multimodal framework, radiotherapy complements surgery and systemic therapy to optimise disease control while preserving function [5,6,7,8].

### 4.5. Clinical Implications and Future Perspectives

The therapeutic evolution of BCC exemplifies the broader shift from a purely surgical paradigm toward multimodal precision oncology in cutaneous malignancies. While surgery remains central to the management of most BCCs, the availability of targeted and immunomodulatory therapies has transformed the care of advanced disease, expanding treatment options and enabling individualized, stage-adapted management strategies [5,6,7,8].

## 5. Cutaneous Squamous Cell Carcinoma: Integrating High-Risk Surgery, Radiotherapy, and Immunotherapy

### 5.1. Changing Epidemiology and Clinical Challenges of cSCC

Cutaneous squamous cell carcinoma (cSCC) is the second most common skin cancer, with a steadily rising incidence worldwide. Although the majority of cases are cured surgically, a clinically significant subset behaves aggressively, with increased risks of local recurrence, nodal metastasis, and disease-specific mortality. This risk is particularly pronounced in elderly patients, immunosuppressed individuals, and tumours arising in the head and neck region.

Advances in cutaneous oncology have reframed cSCC as a biologically heterogeneous disease, requiring individualized risk stratification and multimodal management rather than uniform surgical excision [9,10,11,12,13].

### 5.2. Surgical Innovation in High-Risk cSCC

Surgery remains the cornerstone of curative treatment in cSCC. However, high-risk tumours—defined by depth of invasion, poor differentiation, perineural invasion, recurrence, tumour size, or critical anatomic location—require advanced surgical strategies beyond standard excision.

Mohs micrographic surgery has assumed an increasingly important role in high-risk cSCC, providing superior margin control compared with conventional excision, particularly for tumours with ill-defined borders, recurrent disease, perineural involvement, and lesions located in facial and auricular regions. Innovations in surgical planning also include systematic assessment of perineural spread, selective use of preoperative imaging, and reconstructive strategies designed to anticipate adjuvant radiotherapy [9,10,11,12,13].

### 5.3. Perineural Disease and Advanced Surgical Considerations

Perineural invasion (PNI) represents one of the most adverse prognostic features in cSCC. Its detection has improved through heightened histopathologic awareness and advances in imaging, particularly magnetic resonance imaging for clinically significant nerve involvement.

Surgical innovation in this context includes extended margin control along affected nerve pathways, close collaboration with head and neck surgical teams, and early integration of adjuvant radiotherapy. Collectively, these approaches reflect a shift from localized excision toward regional oncologic surgery tailored to tumour biology and patterns of spread [9,10,11,12,13].

### 5.4. Adjuvant Radiotherapy: Refining Indications

Radiotherapy has long played a role in cSCC management, but recent innovations have refined its indications. Rather than routine use, adjuvant radiotherapy is now reserved for well-defined high-risk scenarios, including extensive perineural invasion, close or positive margins when re-excision is not feasible, deep invasion beyond subcutaneous tissue, and selected patterns of nodal disease.

Improved coordination of surgical and radiotherapeutic planning has enhanced oncologic outcomes while minimising functional morbidity, particularly in anatomically sensitive regions [9,10,11,12,13].

### 5.5. Immunotherapy and the Evolving Surgical Paradigm

The advent of immune checkpoint inhibitors, particularly anti–PD-1 agents, has fundamentally altered the management of advanced cSCC. Durable responses have been observed in patients with locally advanced or metastatic disease who were previously considered unsuitable for curative local therapy.

From a surgical perspective, immunotherapy has introduced several novel strategies, including conversion of unresectable tumours into resectable disease following systemic response, consolidative surgery to remove residual disease after partial response, and salvage surgery for localized progression during otherwise controlled systemic treatment. This dynamic interaction between immunotherapy and surgery underscores the importance of continuous reassessment and flexible treatment sequencing.

Beyond advances in margin-controlled surgery and reconstructive techniques, the integration of systemic immunotherapy—particularly the anti–PD-1 antibody cemiplimab—has expanded the role of the dermatologic surgeon within a multidisciplinary framework. Surgery, systemic therapy, and, in selected cases, radiotherapy are now strategically combined to optimise disease control while preserving function. Although evidence supporting immunotherapy in rare adnexal tumours remains limited, these developments exemplify the broader transition toward integrated precision oncology in cutaneous cancer care [9,10,11,12,13].

### 5.6. cSCC in Immunosuppressed Patients

Immunosuppressed populations, particularly solid-organ transplant recipients, represent a distinct clinical challenge in cSCC management. Tumours in this setting tend to be more aggressive, multifocal, and recurrent, often necessitating repeated surgical interventions.

Innovations in care include intensified surveillance protocols, early use of margin-controlled surgery, close coordination with transplant teams to optimise immunosuppressive regimens, and cautious consideration of systemic therapies, balancing oncologic benefit against graft survival. These strategies highlight the necessity of personalised, multidisciplinary care pathways for complex cSCC [9,10,11,12,13].

## 6. Melanoma: From Wide Excision to Integrated Systemic and Surgical Care

### 6.1. Surgery as the Backbone of Melanoma Management

Wide local excision remains essential for local control of primary melanoma, while sentinel lymph node biopsy (SLNB) continues to provide critical staging and prognostic information. Refinements in surgical management include more selective criteria for SLNB, improved patient selection based on tumour thickness and risk factors, and the selective incorporation of imaging modalities to complement nodal assessment in clinically complex cases.

Overall, the surgical management of melanoma has evolved toward greater precision, aiming to minimise morbidity without compromising oncologic outcomes. This evolution reflects a broader shift toward risk-adapted surgery integrated within multidisciplinary treatment pathways [14,15,16,17,18,19,20].

### 6.2. Neoadjuvant Systemic Therapy and Surgical Implications

Neoadjuvant systemic therapy represents one of the most transformative developments in contemporary melanoma management. Immune checkpoint inhibitors targeting the programmed cell death pathway—most notably the anti–PD-1 antibodies nivolumab and pembrolizumab, used either as monotherapy or in combination with the anti–CTLA-4 agent ipilimumab—have demonstrated high pathological response rates and durable disease control in selected patients with resectable stage III and oligometastatic stage IV melanoma.

In parallel, molecularly targeted therapies directed against the MAPK pathway have expanded neoadjuvant options for patients harbouring BRAF V600 mutations. Combinations of BRAF inhibitors (dabrafenib, vemurafenib, encorafenib) with MEK inhibitors (trametinib, cobimetinib, binimetinib) offer rapid tumour regression and may facilitate surgical resection in selected cases, particularly when prompt disease control is required.

From a surgical perspective, neoadjuvant systemic therapy introduces both significant opportunities and technical challenges. Tumour burden reduction may facilitate less extensive surgery and improved functional outcomes, while pathological response has emerged as a powerful surrogate marker of prognosis, informing postoperative risk stratification and personalisation of adjuvant therapy. Conversely, therapy-induced changes—including inflammation, fibrosis, altered tissue planes, and nodal architectural distortion—require adaptation of surgical technique, careful timing of intervention, and meticulous preoperative planning. These considerations highlight the importance of close multidisciplinary coordination throughout neoadjuvant treatment and subsequent surgical management [14,15,16,17,18,19,20].

While pathologic complete response (pCR) has emerged as a promising surrogate marker of prognosis in the neoadjuvant melanoma setting, its interpretation requires caution. Although pCR is associated with favorable relapse-free survival, it does not uniformly translate into long-term cure, nor does it eliminate the need for surgical resection. Moreover, neoadjuvant therapy may increase surgical complexity due to inflammation, fibrosis, altered tissue planes, and potential wound-healing complications. These factors underscore the importance of careful patient selection, timing of surgery, and close multidisciplinary coordination.

### 6.3. Surgery in Advanced and Oligometastatic Melanoma

Despite major advances in systemic therapy, surgery retains an important role in advanced melanoma. Indications include management of oligometastatic disease, treatment of oligoprogression during otherwise effective immunotherapy or targeted therapy, and control of symptomatic local complications.

In the era of highly effective immune checkpoint inhibitors and BRAF–MEK targeted combinations, surgery is increasingly deployed as a complementary, strategically timed intervention rather than a standalone curative modality. When appropriately integrated into systemic treatment pathways, surgical resection may extend disease control, delay progression, and preserve quality of life in selected patients [14,15,16,17,18,19,20].

## 7. Merkel Cell Carcinoma and Rare Cutaneous Malignancies

### 7.1. Merkel Cell Carcinoma: A Model of Integrated Oncologic Care

Merkel cell carcinoma (MCC) exemplifies the need for fully integrated oncologic care in cutaneous malignancies. Even when localized disease is amenable to complete surgical excision, the risk of local, regional, and distant recurrence remains high. As a result, adjuvant radiotherapy is frequently indicated to improve locoregional control, particularly in primary tumours with adverse prognostic features or nodal involvement.

The introduction of immune checkpoint inhibitors has profoundly transformed outcomes in advanced MCC, leading to durable responses and prolonged disease control in a substantial proportion of patients. In this context, surgery increasingly functions as part of a coordinated, multimodal strategy, including initial tumour resection, consolidation of residual disease following systemic response, and management of isolated progression or symptomatic lesions.

In MCC, surgical innovation lies not primarily in novel operative techniques, but in optimal timing, patient selection, and integration within multidisciplinary treatment pathways. Close coordination between dermatologic surgeons, surgical oncologists, radiation oncologists, and medical oncologists is essential to tailor treatment sequencing and maximise oncologic benefit while preserving function and quality of life [21,22,23,24].

### 7.2. Rare Cutaneous Tumours and Adnexal Malignancies

Rare adnexal carcinomas and other uncommon cutaneous malignancies present distinct diagnostic and therapeutic challenges due to their heterogeneity and the scarcity of high-level evidence. In the absence of robust clinical trial data, management relies heavily on extrapolation from more common tumour types, expert consensus, and individualised decision-making.

Key innovations in the management of these rare tumours include the use of margin-controlled surgery to ensure complete tumour clearance, systematic multidisciplinary discussion within specialised tumour boards, and personalised treatment strategies informed by tumour biology, anatomic considerations, and patient-related factors. In selected cases, integration of radiotherapy or systemic therapies may be considered, particularly for locally advanced or recurrent disease.

These malignancies underscore the broader principles of precision oncology in dermatology, in which surgical expertise, multidisciplinary collaboration, and adaptive treatment sequencing are essential to optimise outcomes in complex and uncommon clinical scenarios [21,22,23,24].

## 8. Locoregional Therapies in Cutaneous Oncology: Expanding the Surgical Armamentarium

### 8.1. Conceptual Framework

Locoregional therapies occupy an intermediate position between surgery and systemic treatment, offering effective local and regional disease control with reduced morbidity in selected clinical scenarios. Their role has expanded substantially in recent years, particularly in patients with locally advanced disease, multiple or recurrent lesions, limited surgical candidacy, or tumours involving anatomically complex or functionally critical areas.

Rather than competing with dermatologic surgery, locoregional approaches increasingly function as complementary, sequential, or bridging strategies within integrated treatment pathways. Their optimal use depends on careful patient selection, tumour biology, disease extent, and multidisciplinary coordination, reinforcing the concept of precision oncology in cutaneous cancer care [25,26,27].

### 8.2. Electrochemotherapy

Electrochemotherapy (ECT) combines the administration of cytotoxic agents with transient electroporation of cell membranes, enhancing intracellular drug uptake and local cytotoxicity. This technique has demonstrated high response rates in cutaneous and subcutaneous metastases of melanoma, cutaneous squamous cell carcinoma, basal cell carcinoma, and selected rare cutaneous tumours.

From a surgical perspective, ECT represents a meaningful innovation across several clinical contexts. It may serve as a tissue-sparing alternative in anatomically sensitive regions where surgery would result in significant morbidity, as a palliative or cytoreductive option in multifocal or recurrent disease, and as a bridging strategy to surgery in cases where tumour burden reduction is required to enable subsequent resection. Although ECT does not replace surgery, it broadens the therapeutic armamentarium for patients with limited local treatment options and complex disease presentations [25,26,27].

### 8.3. Isolated Limb Perfusion and Infusion

Isolated limb perfusion (ILP) and isolated limb infusion (ILI) represent highly specialised locoregional techniques, primarily indicated for advanced melanoma and selected soft tissue sarcomas with extensive limb involvement. By delivering high-dose chemotherapy to a confined anatomical region while minimising systemic exposure, these approaches can achieve substantial tumour regression and local disease control.

Although resource-intensive and limited to specialised centres, ILP and ILI illustrate the extreme end of locoregional surgical innovation, where aggressive oncologic control is pursued while avoiding radical surgical procedures such as amputation. In carefully selected patients, effective tumour reduction may facilitate subsequent conservative surgery or prolonged limb preservation [25,26,27].

### 8.4. Photodynamic Therapy and Other Locoregional Modalities

Photodynamic therapy (PDT) retains an established role in the management of superficial non-melanoma skin cancers and field cancerization, particularly in patients who are poor surgical candidates or in whom repeated excisions would lead to cumulative morbidity. Advances in photosensitising agents, light delivery systems, and treatment protocols have improved both efficacy and tolerability.

Additional locoregional approaches, including topical immunomodulators and intralesional therapies, further expand the spectrum of minimally invasive interventions available in cutaneous oncology. While generally not curative for advanced disease, these modalities may complement surgical management in selected contexts, contributing to disease control, symptom relief, and preservation of function [25,26,27].

## 9. Diagnostic and Intraoperative Technologies: Refining Precision Surgery

### 9.1. Advances in Preoperative Assessment

Advances in diagnostic imaging and non-invasive assessment have significantly refined preoperative planning in cutaneous oncology. High-frequency ultrasound, reflectance confocal microscopy, and advanced dermatoscopic techniques allow more accurate delineation of tumour margins, depth of invasion, and subclinical extension. These tools are particularly valuable in anatomically sensitive areas, recurrent tumours, and lesions with ambiguous clinical borders.

By improving tumour characterisation before surgery, these technologies support risk-adapted surgical strategies and facilitate more informed decision-making. They assist in identifying patients who may benefit from margin-controlled surgery, neoadjuvant systemic therapy, or multimodal treatment approaches, thereby enhancing precision while minimising unnecessary morbidity [28,29,30,31].

### 9.2. Intraoperative Margin Assessment and Real-Time Decision-Making

Accurate margin assessment remains central to innovation in oncologic surgery. Mohs micrographic surgery represents the gold standard for complete margin control in selected cutaneous malignancies; however, additional advances continue to refine intraoperative assessment. Improvements in frozen section processing, targeted immunohistochemical staining, and the incorporation of digital pathology have enhanced the detection of residual or subclinical tumour involvement.

Emerging technologies aim to reduce operative time, improve sensitivity for tumour spread beyond clinically visible margins, and strengthen real-time communication between surgeons and pathologists. Together, these developments reinforce surgery as a precision discipline, guided by immediate oncologic feedback and iterative intraoperative decision-making [28,29,30,31].

### 9.3. Digital Follow-Up and Longitudinal Monitoring

Digital surveillance systems and sequential imaging have become integral components of modern cutaneous oncology, particularly for patients with multiple tumours, field cancerization, or high risk of recurrence. Digital dermoscopy, total body photography, and structured imaging follow-up enable earlier detection of recurrence or new primary tumours and facilitate timely surgical intervention.

The integration of digital monitoring with surgical care reflects a broader shift toward longitudinal, patient-centred oncology, in which disease management extends beyond the operative episode. By supporting continuous risk assessment and adaptive treatment planning, these technologies complement precision surgery and contribute to improved long-term outcomes [28,29,30,31].

Despite the rapid expansion of artificial intelligence-based diagnostic tools, several limitations remain. Many AI systems lack external validation, long-term outcome data, and regulatory approval for routine clinical use. Concerns include algorithmic bias, overfitting to selected datasets, and the risk of over-reliance on automated outputs without adequate clinical oversight. AI technologies should therefore be regarded as decision-support tools rather than substitutes for expert clinical judgment, particularly in oncologic decision-making.

## 10. Therapeutic Algorithms and Integrated Decision-Making

### 10.1. From Linear to Adaptive Treatment Algorithms

Traditional treatment algorithms in cutaneous oncology were largely linear and surgery-centred, with subsequent therapies introduced only in cases of recurrence or progression. Recent innovations have transformed these static pathways into adaptive and dynamic algorithms that integrate tumour biology, disease extent, patient-related factors, and response to therapy over time [Table 2].

In contemporary practice, surgical intervention may precede, follow, or be intercalated with systemic therapy depending on disease stage, treatment response, and functional considerations. Locoregional treatments may serve as primary modalities, adjuncts to surgery or systemic therapy, or salvage options in cases of limited progression or symptomatic disease. Decision-making is increasingly multidisciplinary and iterative, with treatment plans reassessed at key clinical milestones rather than fixed at initial diagnosis.

This shift toward adaptive algorithms reflects the growing complexity of cutaneous oncology and the need for flexible strategies capable of responding to evolving disease behaviour [28,29,30,31].

### 10.2. The Evolving Role of Dermatologic Surgeons in Multimodal Care

Within this integrated framework, dermatologic surgeons now play a pivotal role that extends well beyond tumour excision. Their expertise is central to treatment sequencing decisions, evaluation of response to systemic therapies, identification of optimal timing for surgical intervention, and management of treatment-related complications, including those associated with immunotherapy, targeted therapy, and radiotherapy.

Dermatologic surgeons also contribute to long-term surveillance planning, balancing oncologic vigilance with functional preservation and quality-of-life considerations. This expanded role underscores the importance of surgical leadership within multidisciplinary oncology teams and highlights the surgeon’s position at the interface between local and systemic care [28,29,30,31].

## 11. Future Perspectives in Cutaneous Oncology and Dermatologic Surgery

The future of cutaneous oncology is increasingly defined by precision, personalisation, and integration across therapeutic modalities. Advances in molecular profiling, predictive biomarkers, and artificial intelligence–driven diagnostic tools are expected to further refine patient selection for both surgical and non-surgical interventions. These developments will enable more accurate risk stratification, improved prediction of treatment response, and more nuanced therapeutic sequencing.

From a surgical perspective, innovation will continue to focus on maximising oncologic control while minimising morbidity, integrating seamlessly with systemic and locoregional therapies, and preserving function, aesthetics, and quality of life. Technological advances in preoperative assessment, intraoperative guidance, and postoperative surveillance will further support precision oncology surgical decision-making.

As therapeutic options expand and treatment pathways become increasingly complex, the importance of expert clinical judgment will grow. Multidisciplinary collaboration, adaptive treatment algorithms, and longitudinal patient-centred care will be essential to translate scientific innovation into meaningful clinical benefit. In this evolving landscape, dermatologic surgeons will remain central to integrating emerging technologies and therapies into cohesive, individualised care strategies [28,29,30,31].

## 12. Conclusions

Innovations in cutaneous oncology have reshaped dermatologic surgery from a standalone intervention into a central component of integrated, precision oncology cancer care. While surgery remains indispensable for achieving cure and durable local control, its greatest impact increasingly lies in strategic integration with targeted therapies, immunotherapy, radiotherapy, and locoregional techniques.

Across major cutaneous malignancies, advances in margin-controlled surgery, systemic treatments, and diagnostic technologies have enabled adaptive, biology-driven treatment pathways that prioritise oncologic outcomes while preserving function and quality of life. This evolution reflects a broader transformation in oncology—one that values flexibility, personalisation, and patient-centred outcomes over rigid, linear treatment models.

As therapeutic options continue to expand, the future of cutaneous oncology will be defined by precision, adaptability, and multidisciplinary integration. Dermatologic surgeons will play an increasingly central role in coordinating treatment sequencing, interpreting response to systemic therapies, and determining the optimal timing and extent of surgical intervention. By integrating surgical expertise with systemic, radiotherapeutic, and locoregional strategies, clinicians can deliver personalised care that maximises oncologic control while preserving function and quality of life.

## Figures and Tables

**Table 1:** Risk-Adapted Management Framework for Basal Cell Carcinoma.

Tumor Risk Category	Surgical Approach	Role of Mohs Surgery	Adjunctive Radiotherapy	Systemic Therapy Considerations
Low-risk BCC	Standard excision (4–5 mm margins)	Generally not required	Rarely indicated	Not indicated
High-risk BCC (anatomic location, aggressive histology, recurrent)	Margin-controlled surgery	Preferred in high-risk and facial tumors	Consider in perineural invasion, positive margins	Not indicated unless locally advanced
Locally advanced BCC	Individualized surgical planning	Case-dependent	May be indicated	Hedgehog inhibitors (vismodegib, sonidegib); PD-1 inhibitor (cemiplimab)
Metastatic BCC	Surgery rarely curative	Not applicable	Palliative role	Hedgehog inhibitors or immunotherapy

**Table 2:** Integrated therapeutic algorithms in major cutaneous malignancies.

Tumor Type	Standard Surgical Approach	When Systemic Therapy Is Integrated	Role of Radiotherapy/Locoregional Therapy	Surgical Role in Advanced Disease
Basal cell carcinoma (BCC)	Conventional excision or Mohs micrographic surgery	Hedgehog pathway inhibitors (vismodegib, sonidegib); PD-1 blockade after HHI failure	Radiotherapy when surgery is contraindicated; electrochemotherapy (ECT) in selected cases	Neoadjuvant downstaging; definitive margin-controlled clearance
Cutaneous squamous cell carcinoma (cSCC)	Excision or Mohs surgery for high-risk tumors	PD-1 inhibitors (cemiplimab, pembrolizumab) for locally advanced/metastatic disease	Adjuvant radiotherapy for perineural invasion or close/positive margins; ECT for selected lesions	Salvage, consolidative, or response-adapted surgery
Melanoma	Wide local excision ± sentinel lymph node biopsy (SLNB)	Neoadjuvant/adjuvant immunotherapy or BRAF–MEK targeted therapy	Radiotherapy for selected nodal/CNS disease; isolated limb perfusion/infusion (ILP/ILI)	Resection of oligometastatic disease; control of oligoprogression
Merkel cell carcinoma (MCC)	Excision with margin control ± SLNB	PD-1/PD-L1 inhibitors for advanced disease	Adjuvant radiotherapy frequently recommended for locoregional control	Selected consolidative or salvage resections
Rare adnexal tumors	Margin-controlled surgery (preferably Mohs or staged excision)	Case-based systemic therapy guided by histology and molecular profile	Radiotherapy or locoregional approaches as adjuncts in selected cases	Individualized multidisciplinary decision-making

Abbreviations: ECT, electrochemotherapy; PNI, perineural invasion; CNS, central nervous system; ILP, isolated limb perfusion; HHIs, Hedgehog pathway inhibitors; PD-1, programmed cell death protein 1; PD-L1, programmed death-ligand 1; SLNB, sentinel lymph node biopsy; RT, radiotherapy.

## Data Availability

No new data were generated or analysed in this study. This article is a narrative review based exclusively on previously published literature.

## References

[1] Garbe C., Amaral T., Peris K., Hauschild A., Arenberger P., Bastholt L., Bataille V., del Marmol V., Dréno B., Fargnoli M.C. (2020). European consensus-based interdisciplinary guideline for melanoma. Part 1: Diagnostics—Update 2019. Eur. J. Cancer.

[2] Stratigos A.J., Garbe C., Dessinioti C., Lebbe C., Bataille V., Bastholt L., Dreno B., Fargnoli M.C., Forsea A.M., Frenard C. (2020). European interdisciplinary guideline on invasive squamous cell carcinoma of the skin: Part 2. Treatment. Eur. J. Cancer.

[3] Peris K., Fargnoli M.C., Kaufmann R., Arenberger P., Bastholt L., Seguin N.B., Bataille V., Brochez L., del Marmol V., Dummer R. (2023). European consensus-based interdisciplinary guideline for diagnosis and treatment of basal cell carcinoma—Update 2023. Eur. J. Cancer.

[4] Schmults C.D., Blitzblau R., Aasi S.Z., Alam M., Amini A., Bibee K., Bordeaux J., Chen P.L., Contreras C.M., DiMaio D. (2023). Basal Cell Skin Cancer, Version 2.2024, NCCN Clinical Practice Guidelines in Oncology. J. Natl. Compr. Canc. Netw..

[5] Sekulic A., Migden M.R., Oro A.E., Dirix L., Lewis K.D., Hainsworth J.D., Solomon J.A., Yoo S., Arron S.T., Friedlander P.A. (2012). Efficacy and safety of vismodegib in advanced basal-cell carcinoma. N. Engl. J. Med..

[6] Migden M.R., Guminski A., Gutzmer R., Dirix L., Lewis K.D., Combemale P., Herd R.M., Kudchadkar R., Trefzer U., Gogov S. (2015). Treatment with two different doses of sonidegib in patients with locally advanced or metastatic basal cell carcinoma (BOLT): A multicentre, randomised, double-blind phase 2 trial. Lancet Oncol..

[7] Dummer R., Ascierto P.A., Basset-Seguin N., Dréno B., Garbe C., Gutzmer R., Hauschild A., Krattinger R., Lear J., Malvehy J. (2020). Sonidegib and vismodegib in the treatment of patients with locally advanced basal cell carcinoma: A joint expert opinion. J. Eur. Acad. Dermatol. Venereol..

[8] Stratigos A.J., Sekulic A., Peris K., Bechter O., Prey S., Kaatz M., Lewis K.D., Basset-Seguin N., Chang A.L.S., Dalle S. (2021). Cemiplimab in locally advanced basal cell carcinoma after hedgehog inhibitor therapy: An open-label, multi-centre, single-arm, phase 2 trial. Lancet Oncol..

[9] Winge M.C.G., Kellman L.N., Guo K., Tang J.Y., Swetter S.M., Aasi S.Z., Sarin K.Y., Chang A.L.S., Khavari P.A. (2023). Advances in cutaneous squamous cell carcinoma. Nat. Rev. Cancer.

[10] Migden M.R., Rischin D., Schmults C.D., Guminski A., Hauschild A., Lewis K.D., Chung C.H., Hernandez-Aya L.F., Lim A.M., Chang A.L.S. (2018). PD-1 blockade with cemiplimab in advanced cutaneous squamous-cell carcinoma. N. Engl. J. Med..

[11] Grob J.J., Gonzalez R., Basset-Seguin N., Vornicova O., Schachter J., Joshi A., Meyer N., Grange F., Piulats J.M., Bauman J.R. (2020). Pembrolizumab Monotherapy for Recurrent or Metastatic Cutaneous Squamous Cell Carcinoma: A Single-Arm Phase II Trial (KEYNOTE-629). J. Clin. Oncol..

[12] Pei M., Wiefels M., Harris D., Torres J.M.V., Gomez-Fernandez C., Tang J.C., Aya L.H., Samuels S.E., Sargi Z., Weed D. (2024). Perineural invasion in head and neck cutaneous squamous cell carcinoma. Cancers.

[13] Mittal A., Colegio O.R. (2017). Skin cancers in organ transplant recipients. Am. J. Transpl..

[14] Rashid S., Shaughnessy M., Tsao H. (2023). Melanoma classification and management in the era of molecular medicine. Dermatol. Clin..

[15] Rozeman E.A., Menzies A.M., van Akkooi A.C.J., Adhikari C., Bierman C., A van de Wiel B., A Scolyer R., Krijgsman O., Sikorska K., Eriksson H. (2019). Identification of the optimal combination dosing schedule of neoadjuvant ipilimumab plus nivolumab in macroscopic stage III melanoma (OpACIN-neo): A multicentre, phase 2, randomised, controlled trial. Lancet Oncol..

[16] Reijers I.L.M., Menzies A.M., van Akkooi A.C.J., Versluis J.M., Heuvel N.M.J.v.D., Saw R.P.M., Pennington T.E., Kapiteijn E., van der Veldt A.A.M., Suijkerbuijk K.P.M. (2022). Personalized response-directed surgery and adjuvant therapy after neoadjuvant ipilimumab and nivolumab in high-risk stage III melanoma: The PRADO trial. Nat. Med..

[17] Rozeman E.A., Hoefsmit E.P., Reijers I.L.M., Saw R.P.M., Versluis J.M., Krijgsman O., Dimitriadis P., Sikorska K., van de Wiel B.A., Eriksson H. (2021). Survival and biomarker analyses from OpACIN-neo. Nat. Med..

[18] Amaria R.N., Prieto P.A., Tetzlaff M.T., Reuben A., Andrews M.C., I Ross M., Glitza I.C., Cormier J., Hwu W.-J., A Tawbi H. (2018). Neoadjuvant dabrafenib and trametinib in high-risk melanoma. Lancet Oncol..

[19] Teixido C., Castillo P., Martinez-Vila C., Arance A., Alos L. (2021). Molecular markers and targets in melanoma. Cells.

[20] Keller H.R., Hanes D.A., McCabe J.K., Lascano D., Shin P., Goldfarb M., Essner R. (2025). Metastatic melanoma outcomes and the evolving role of surgery in the immunotherapy era. JAMA Surg..

[21] Tai P., Alqaisi O., Al-Ghabeesh S., Sijarina L., Yu E., Thachuthara A.J., Assouline A., Souied O., Hagel K., Joseph K. (2025). Immune Checkpoint Inhibitors in Merkel Cell Carcinoma of the Skin: A 2025 Comprehensive Review. Cancers.

[22] Lewis D.J., Sobanko J.F., Etzkorn J.R., Shin T.M., Giordano C.N., McMurray S.L., Walker J.L., Zhang J., Miller C.J., Higgins H.W. (2023). Merkel cell carcinoma. Dermatol. Clin..

[23] Zaggana E., Konstantinou M.P., Krasagakis G.H., de Bree E., Kalpakis K., Mavroudis D., Krasagakis K. (2022). Merkel Cell Carcinoma—Update on Diagnosis, Management and Future Perspectives. Cancers.

[24] Seger E.W., Neill B.C., Tolkachjov S.N. (2023). Adnexal and sebaceous carcinomas. Dermatol. Clin..

[25] Rotunno R., Campana L.G., Quaglino P., de Terlizzi F., Kunte C., Odili J., Gehl J., Ribero S., Liew S., Marconato R. (2018). Electrochemotherapy of unresectable cutaneous tumours with reduced dosages of intravenous bleomycin: Analysis of 57 patients from the International Network for Sharing Practices of Electrochemotherapy registry. J. Eur. Acad. Dermatol. Venereol..

[26] O’Donoghue N., Mowatt D., Sykes A.J. (2019). Electrochemotherapy and ablative therapies in non-melanoma skin cancer. Clin. Oncol..

[27] Davies E.J., Reijers S.J.M., Van Akkooi A.C.J., Van Houdt W., Hayes A. (2022). Isolated limb perfusion for locally advanced melanoma in the immunotherapy era. Eur. J. Surg. Oncol..

[28] Navarrete-Dechent C., Cordova M., Aleissa S., Kose K., Lee E.H., Rossi A.M., Nehal K.S. (2020). Reflectance confocal microscopy navi-gation. J. Am. Acad. Dermatol..

[29] Wortsman X. (2023). Top advances in dermatologic ultrasound. J. Ultrasound Med..

[30] Marra A., Morganti S., Pareja F., Campanella G., Bibeau F., Fuchs T., Loda M., Parwani A., Scarpa A., Reis-Filho J. (2025). Artificial intelligence entering the pathology arena in oncology: Current applications and future perspectives. Ann. Oncol..

[31] Abdollahimajd F., Abbasi F., Motamedi A., Koohi N., Robati R.M., Gorji M. (2025). Using the power of artificial intelligence to improve the diagnosis and management of nonmelanoma skin cancer. J. Res. Med. Sci..

